# The impact of mosquito proof lids of underground tanks “*tanka*” on the breeding of *Anopheles stephensi* in a village in western Rajasthan, India

**DOI:** 10.1186/s12936-021-03939-0

**Published:** 2021-10-19

**Authors:** Himmat Singh, Sanjeev Kumar Gupta, Kumar Vikram, Rekha Saxena, Amit Sharma

**Affiliations:** 1 ICMR-National Institute of Malaria Research, Sector 8 Dwarka, Delhi, 110077 India; 2 Indian Council of Medical Research, V. Ramalingaswami Bhawan, Ansari Nagar, P.O. Box No. 4911, New Delhi, 110029 India

**Keywords:** Malaria, *Tankas*, Western Rajasthan, *Anopheles stephensi*

## Abstract

**Background:**

Western Rajasthan in India has a typical desert climate. Until the introduction of the canal water irrigation system, malaria was an unstable and seasonal occurrence. Due to the scarcity of water, the community practised having one large underground tank (locally known as the *tanka*) in their house to collect rainwater for long-term household use. *Anopheles stephensi,* one of the major malaria vectors, breeds in improperly covered "*tankas*” if not properly covered and harbours a vector population throughout the year.

**Methods:**

Two villages, Ajasar (intervention) and Tota (control), with similar ecological features, were selected for the study. A pre-intervention survey was carried out in both villages to assess the presence and quality of lids of *tankas*, and mosquito breeding and adult mosquito density. Awareness of the community about malaria and mosquitoes was also assessed during the pre-intervention period. In the intervention village, damaged or improper lids were replaced with improved mosquito proof polyvinyl chloride plastic (PVC) lids and lasted longer than the conventional lids. The fitness of the lids was assessed one year after the pre-intervention survey. The entomological assessment was carried out in both intervention and non-intervention villages. The level of community awareness about malaria, mosquitoes, their breeding places, and the role of *tankas* in malaria transmission was assessed both during pre- and post-intervention.

**Results:**

During the pre-intervention survey**,** *Anopheles* breeding was found in 22.1% (58/262) of *tankas* in the intervention village and 27.1% (19/70) in *tankas* in the control village. Mosquito breeding was observed in the *tankas* with iron lids in the intervention village (48.3%) and the control village (42.1%). In the intervention village, out of 262 *tankas* in the village, 200 lids were replaced, resulting in the complete absence of mosquito breeding.

In the pre-intervention survey conducted in May 2018, *Anopheles stephensi* consisted of 46% of adult mosquitoes in the intervention village and 55% in the control village. Its density was significantly reduced to 0.55 per man-hour (94.95%) and 0.22 per man-hour (97.8%) in the post-intervention survey in June 2018 and a follow-up survey in May 2019, respectively, in the intervention village.

**Discussion:**

The density of *Anopheles stephensi* adults was reduced significantly (97.8%) in the intervention village due to complete prevention of breeding in the underground *tankas* in the intervention village as compared to the control with no density reduction. The awareness level of the community was also improved due to their involvement in the study.

**Conclusion:**

Provision of proper metal lids or replacement of damaged lids on underground water storage tanks as an environmental management approach prevented the breeding of  the malaria vector, *Anopheles stephensi,* in a desert village in western Rajasthan.

## Background

The arid and semi-arid regions of western India are prone to malaria outbreaks [1]. Major malaria epidemics have been reported in these regions where malaria incidence is influenced by the amount of rainfall [2, 3]. However, after the establishment of the Indira Gandhi Canal for catering water in western Rajasthan in 1970, malaria became a major problem [4, 5]. The ecological scenario in terms of cropland and vegetated areas increased by ~ 68% due to increased water availability [6] The breeding of malaria vectors in western Rajasthan was also aided by erratic rainfalls, migration and community water storage activities. The underground water tanks (locally known as the *tankas*) are the primary water storage containers in almost every house in the districts of Jodhpur, Jaisalmer, Barmer and Bikaner in Western Rajasthan. *Tanka* is an underground rainwater collection usually covered and impermeable cistern on shallow ground. *Tankas* collect rainwater from rooftops, courtyards, and artificially constructed catchments [7].

Malaria transmission in the *Thar* desert of India is primarily caused by *Anopheles stephensi*, which breeds almost exclusively in the underground water reservoirs, such as ‘*tanka*’ and ‘*beri*’ (step wells) [8, 9]. According to some studies, the *tankas* become potential breeding grounds for the main malaria vector *Anopheles stephensi* due to damaged lids with holes due to rusting, broken lids, dents and gaps between lids and frames due to bends [9, 10]. During the summer season (April-June), in harsh climatic conditions, *Anopheles* larvae are confined to the *tankas* with no or damaged lids. In monsoon and post-monsoon seasons, *Anopheles* mosquitoes cater in the rainwater collections, while the *tankas* serve as the *mother foci* for mosquito breeding in lean seasons [7].

The present study aimed to assess the impact on the prevention of mosquito breeding by improving the lid design from traditionally used materials to polyvinyl chloride (PVC) plastic and installing proper lids on open *tankas*. This improved lid type was expected to prevent mosquito entry into *tankas* and to prevent the breeding of mosquitoes, especially of the vector of malaria, *Anopheles stephensi,* in the study village.

## Methods

### Study site

**Fig. 1:**
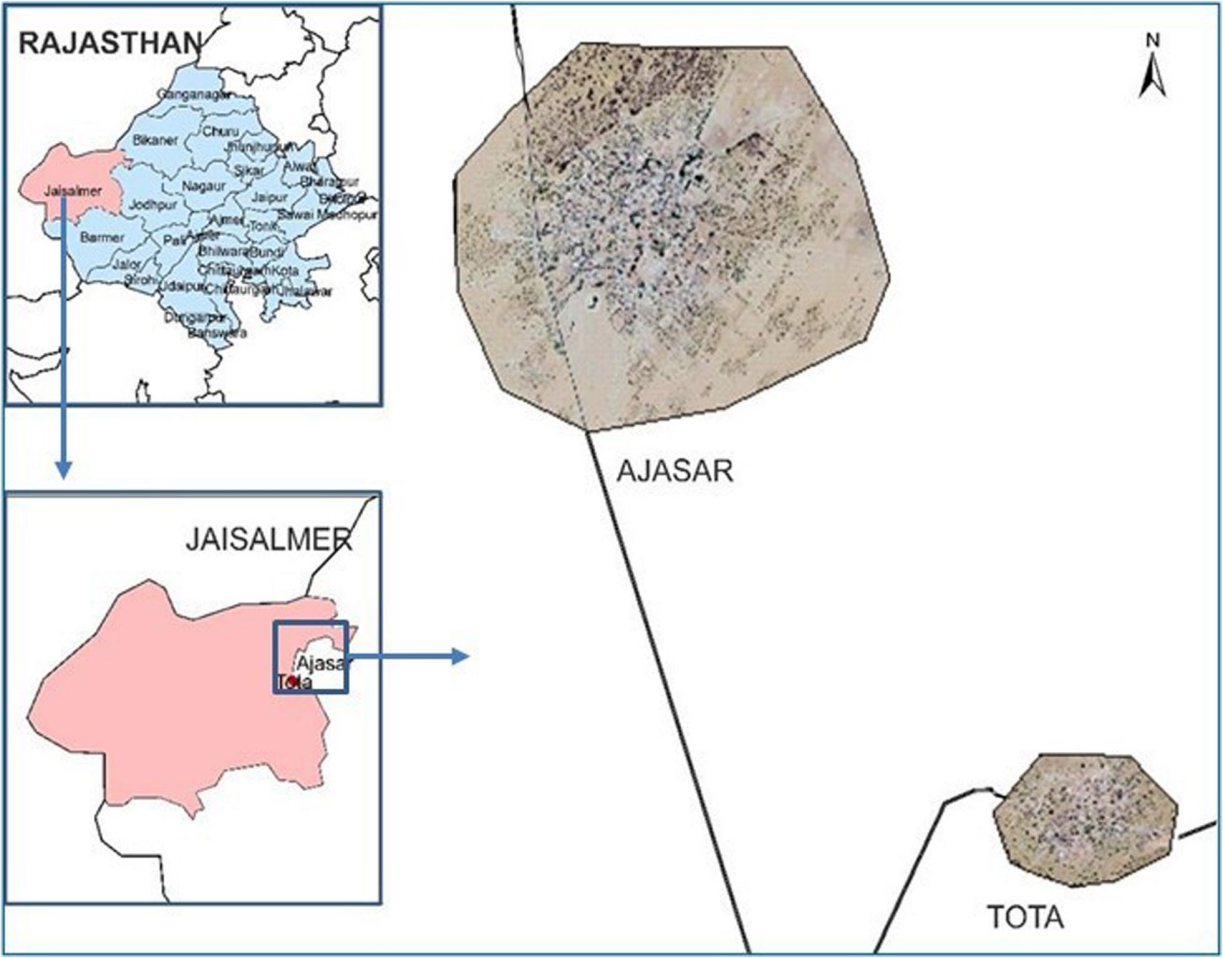
Map showing study areas of Jaisalmer district in Rajasthan, India

Jaisalmer district is located in the *Thar* desert of India between 26°4’ and 28°23′ North Latitude and 69°20′ and 72°42′ East Longitude, is the largest district of Rajasthan and the third largest in India. It shares an international boundary in the west with Pakistan and shares district boundaries with Bikaner, Jodhpur and Barmer districts. Among the four blocks of Jaisalmer district i.e. Jaisalmer, Pokaran, Fatehgarh, and Bhaniyana, Pokaran was most affected by malaria [11]. Therefore, Ajasar village in the Pokaran block, with a population of ~ 1500 people, was chosen for the study having malaria incidence of  > 5 cases/1000 (Annual Parasite Index) after consulting with the Medical Officer of the Community Health Centre, Pokhran in August 2017. A second village, “Tota”, was included as a non-intervention village having a population of ~ 750, and situated ~ 7 km away from village Ajasar and with similar geographical, ecological and malarious conditions (Fig. 1).

### Entomological monitoring

To evaluate adult and larval mosquito breeding of *Anopheles stephensi, e*ntomological collections were made during the pre-intervention (first fortnight of May 2018), post-intervention (second fortnight of June 2018), and follow-up (May 2019) surveys. Adult mosquitoes were collected using two methods: hand catch and total catch, as per the standard WHO manual [12].

### Aspirator collections

Mosquitoes resting inside human dwellings, cattle sheds and mixed dwellings in the two villages were collected by insect collectors using a flash light and mouth aspirators during the early morning hours (6 to 8 AM). Standard taxonomic keys were used to identify mosquitoes. The man-hour density (PMHD) of each *Anopheles* species was calculated (PMHD = Total no. of mosquitoes collected / No. of person x Time spent in hours).

### Total catches

Mosquitoes resting inside dwelling structures were also collected by the spray sheet collection method, by application of pyrethrum extract using a hand atomizer. Mosquitoes knocked down on white cotton sheets spread out on the floor of the room were picked up 10 min after the spray and transferred to the petri dishes lined with wet cotton or filter paper from sheets spread on the floor and transported to the laboratory.

### Larval collections

Breeding site surveys were conducted in all aquatic habitats within a 1 km radius perimeter of the villages using standard World Health Organization (WHO) larval collection methods. Sampling was done with a dipper of white enamel bowl from *tankas*, clay pot/bird pots, under construction *tankas*, cemented cattle tanks, ground cement tanks and seepage waters. Larvae were identified using standard identification keys after emergence at the adult stage. The breeding sites, such as open cattle tanks and under construction cement tanks, were regularly monitored by the Auxiliary Nurse Midwife for mosquito breeding.

### Intervention with lids

**Fig. 2:**
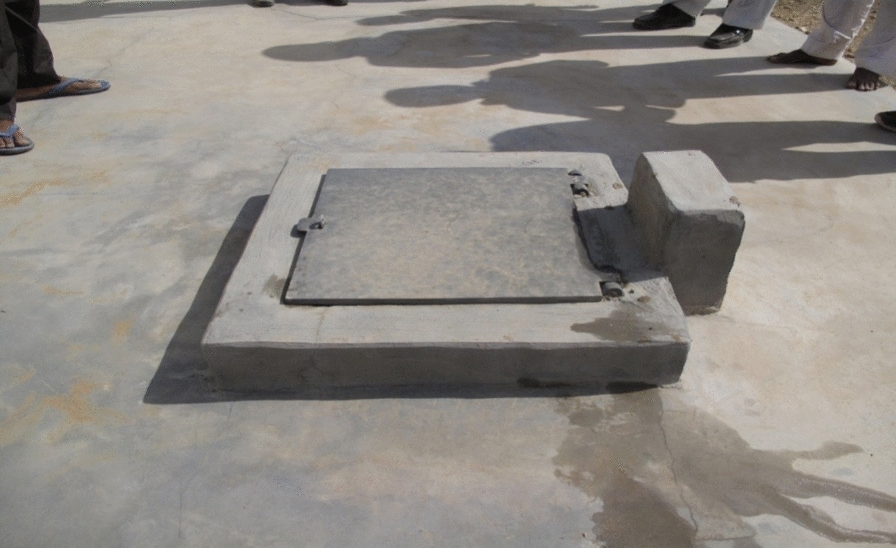
Polyvinyl mosquito proof lid of size 45 × 45 cm

At the time of pre-intervention, there were 262 and 70 *tankas* present in Ajasar and Tota village, respectively**.** Metal lids (dimension 45 cm × 45 cm): a total of 200 (76%) *tankas* were found to be defective due to holes caused by rusting and the gaps between the lids and their frames due to bending or denting. Damaged lids were replaced with the help of villagers and trained skilled persons (the ‘*mistries’*) over a period of 12 days. Consent from house owners in Ajasar village for replacement of lids was taken. Instead of conventional lids made up of iron, aluminium or wood, PVC was used in the modified lids as PVC was considered to be a more durable, rust-proof and economical material. Further steel screws in the hinge joint were used to make the joints rust-free and long-lasting. The frame of the lid was also modified for better binding with the cement properly (Fig. 2).

### Knowledge, attitude and practices (KAP) survey

A door-to-door survey was conducted in 120 households (88 in Ajasar and 32 in Tota village) to assess the knowledge of the community about malaria, mosquitoes, their breeding places, and the role of *tankas* in malaria transmission. The assessment was done before and after the intervention using pre-defined questions. Consent was taken from each participant.

### Information, education and communication (IEC) activities

**Fig. 3:**
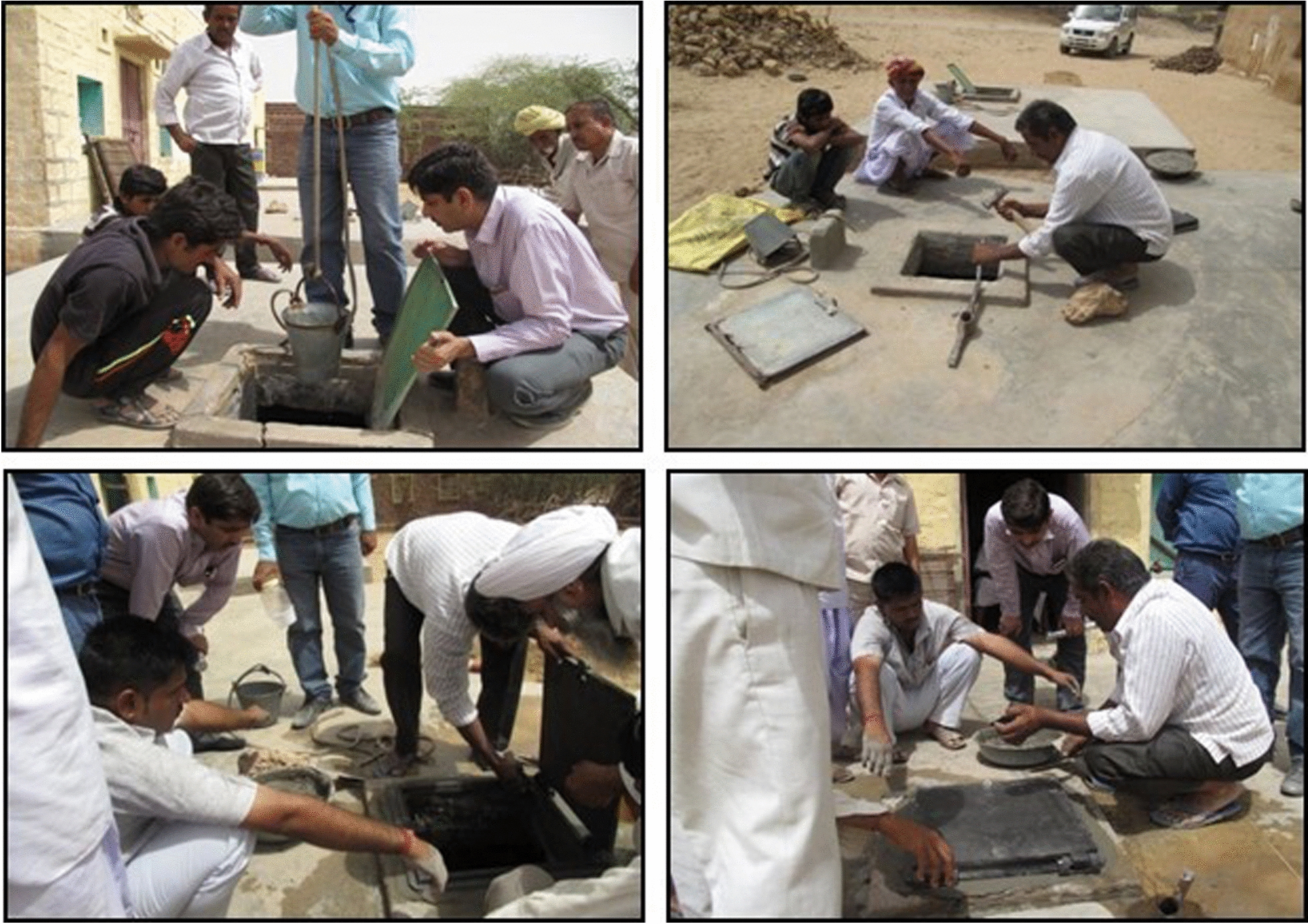
Training/demonstration of process of replacement of *tanka’s* lid

During the pre-intervention phase**,** IEC activities included conducting a door-to-door survey, meeting with the elected village head (*Sarpanch*), Block Development Officer, and community representatives to inform the community about the study’s goal, benefits, and to obtain communal consent for the interventions. Skilled workers *"Mistries"* were also selected from the Ajasar village and trained on the process of replacing lids in the presence of the *Sarpanch* and other important community representatives (Fig. 3).

### Assessing the fitness of tankas

*Tankas* with damaged or missing lids were labelled and geo-referenced using the Global Positioning System (GPS) during the pre-intervention survey for future reference in Ajasar village only. The fitness of the lids, as well as their ability to prevent mosquito breeding, was examined during the follow-up survey after one year. A limitation of the study was the inability to do seasonal studies due to the inadequacy of funds allocated to the study.

## Results

### Pre-intervention survey in study and control villages

In Ajasar village, 54.5% (143/262) *tankas* had lids made of iron sheet, 21% (55/262) of plastic, and 4.2% (11/262) of aluminum or wood, while 18.3% (48/262) *tankas* had no lids. In the control village Tota, 52.8% (37/70) *tankas* had lids made of iron sheet, 27.1% (19/70) of plastic, while 18.5% (13/70) had no lids.

Most of the lids of t*ankas* in both villages were made of the iron sheet – Ajasar 48.3% (28/58); Tota 42.1% (8/19). The proportion of *tankas* with mosquito breeding in these villages was 19.6% and 21.6%, respectively. *Tankas* under construction (60% in the intervention village, 100% in the non-intervention), *tankas* with wooden lids (75% in intervention) and *tankas* without lids (33.3%-intervention, 61.5%-non-intervention) were found to be prone to mosquito breeding (Table 1).

**Table 1:** Impact of replacement of lids on mosquito breeding

Pre-Intervention Assessment (May/2018)				Intervention Phase^a^ (May/2018)		Post-intervention Assessment (June/2019)				Follow-up survey (May/2019)			
Intervention village		Non-Intervention (control village)		Intervention village		Intervention village		Non-Intervention village		Intervention village		Non-Intervention village	
Number of tankas checked	No. positive with larvae (% positivity)	Number of tankas checked	No. with larval breeding (% positivity)	Lid Replaced	Replacement (%)	Number checked	Positive (% positivity)	Number checked	Positive (% positivity)	Number checked	Positive (% positivity)	Number checked	Positive (% positivity)
143	28 (19.6)	37	8 (21.6)	129	90.2	200	0 (%)	31	2 (6.5)	200	4 (2%)	27	9 (33.3)
7	2 (28.6)	0	0 (0.0)	5	71.4			0	0			0	0
4	3 (75.0)	0	0 (0.0)	4	100.0			0	0			0	0
55	6 (10.9)	19	2 (10.5)	14	25.5			16	1 (6.3)			12	1 (8.3)
48	16 (33.3)	13	8 (61.5)	48	100.0			10	2 (20)			7	4 (57.1)
5	3 (60.0)	1	1 (100.0)	0	0.0			5	1 (20)			2	2 (100)
262	58 (22.1)	70	19 (27.1)	200	76.3	200	0 (0%)	62	6 (9.7)	200	4 (2%)	48	16 (33.3)

^a^No replacement of lids in non-intervention village

^b^Follow-up was done only for *tankas* with replaced lids in Ajasar village

In the pre-intervention survey, 22.1% (58/262) *tankas* in the intervention village (Ajasar) and 27.1% (19/70) of *tankas* in the non-intervention village (Tota) were found to have mosquito breeding. The difference between the proportion of the *tankas* with mosquito larvae in the intervention village and the control village was statistically non-significant (z = -0.8814, p = 0.37886 > 0.05, not significant), indicating that there was no difference in larval positivity until the lids were replaced (Table 1).

### Impact of replacement of lids on mosquito breeding

In addition to *tankas,* mosquito larvae were present in seepage water pools, pipeline leakage pools, small pots with water for birds, plastic tanks, desert coolers and cement tanks. In pre-assessment, *Anopheles stephensi* was the predominant species breeding in both intervention and non-intervention villages in the *tankas*, while during both post-intervention and follow-up, breeding of *Anopheles stephensi* was recorded in *tankas* in non-intervention village only. There was no mosquito breeding in other habitats such as clay pots and bird pots in Ajasar village.

### Impact on the density of *Anopheles stephensi*

**Fig. 4:**
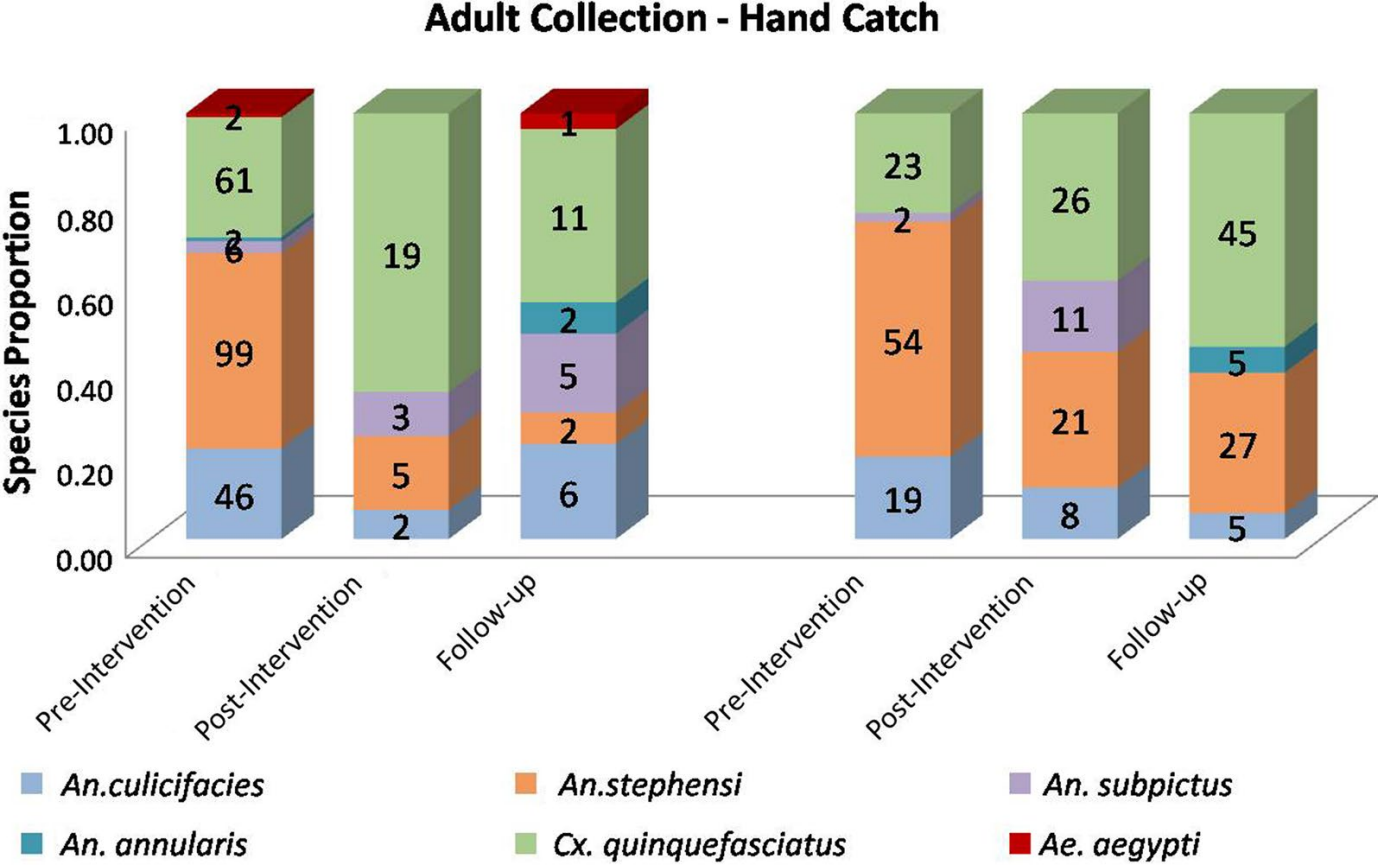
Proportion of adult species collected by hand-catch method in intervention and non-intervention villages before and after replacement of lids

In the pre-intervention survey conducted in May 2019, the proportion of *Anopheles stephensi* was 46% (99/216) and 55% (54/98) in intervention and non-intervention village respectively. Whereas, in the post-intervention and follow-up surveys conducted in 2018 and 2019 respectively, the proportion of *Anopheles stephensi* was reduced significantly to 17% (5/29) [z = 2.925, p = 0.00338, significant at p < 0.05] and 7% (2/27) [z = 3.8197, p = 0.00014, significant at p < 0.05], respectively in the intervention village. In the non-intervention village, the proportion of *Anopheles stephensi* remained predominant 32% (21/66) and 33% (27/82) in the post-intervention and follow-up surveys, respectively (Fig. 4).

**Fig. 5:**
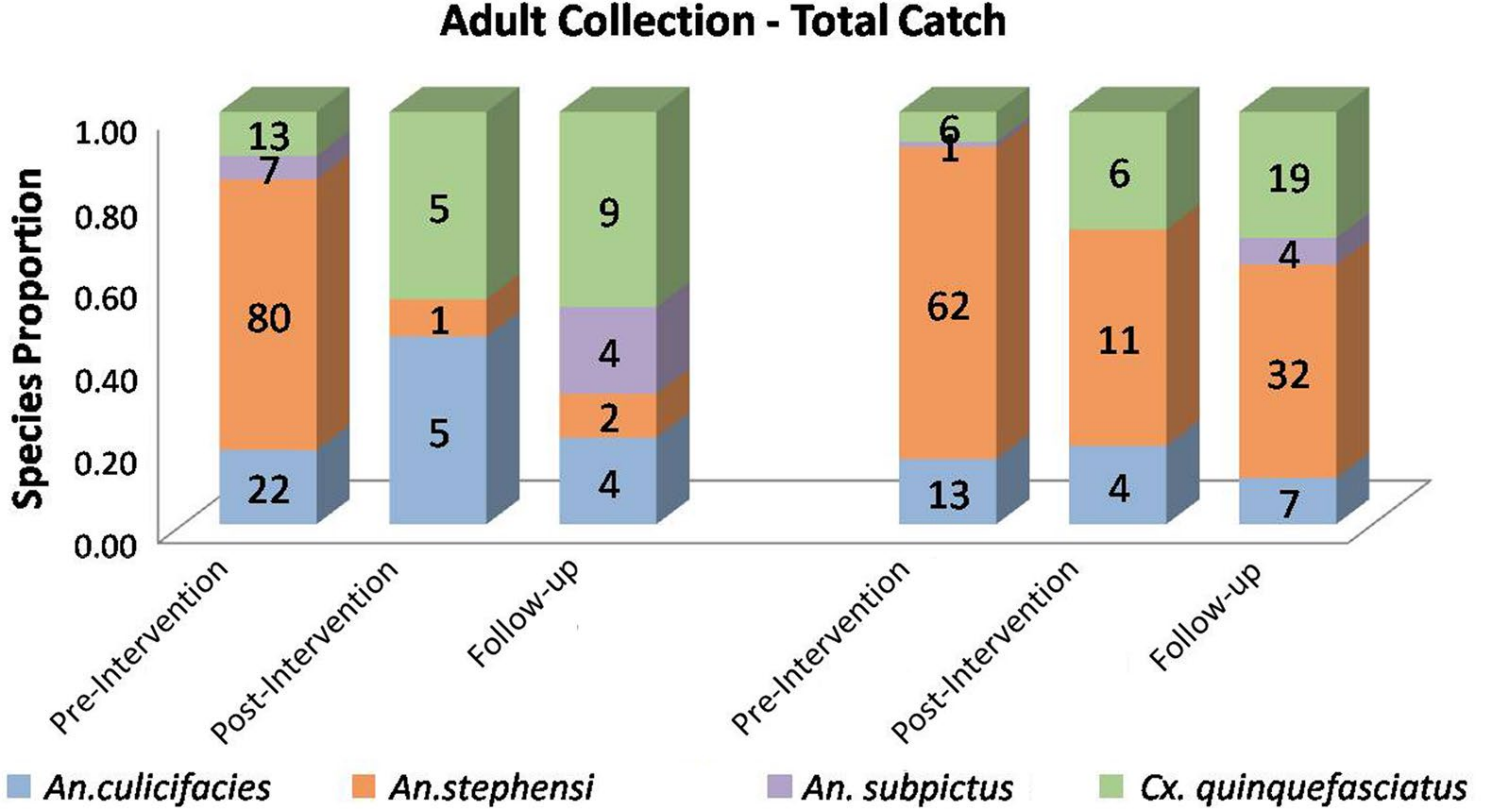
Proportion of adult species collected by total-catch method in intervention and non-intervention villages before and after replacement of lids

Proportion of *Anopheles stephensi* by total-catch method before replacement of lids was found to be 66% (80/122) and 76% (62/82) in intervention and non-intervention villages respectively. While in post-intervention and follow-up surveys in the intervention village, the composition of *Anopheles stephensi* in all adult mosquitoes collected declined significantly to 9% (1/11) [z = 3.6768, p = 0 0.00024, significant at p < 0.05] and 11% (2/19) [z = 4.5245, p < 0.00001, significant at p < 0.05], respectively. *Anopheles stephensi* remained predominant in non-intervention village in the follow-up survey in 2018 (52%; 11/21) and 2019 (52%; 32/62), respectively (Fig. 5).

### Impact of replacing lids on the density of adult mosquitoes

*Anopheles stephensi* had the highest density per man-hour (PMHD) (10.9 and 11.8 in intervention and non-intervention villages, respectively) in pre-intervention assessment, followed by *Anopheles culicifacies* (Table 2). The PMHD of *Anopheles stephensi* in the intervention village reduced significantly to 0.55 (94.95%) in the post-intervention survey and to 0.22 (97.8%) in the further follow-up survey. The reduction of the density of *Anopheles stephensi* in the non-intervention village was moderate at 4.6 (61.1%) and 5.9 (50%) in post-intervention and follow-up surveys, respectively.

**Table 2:** Density per man-hour of *Anopheles* species in the intervention and the non-intervention village before and after replacing the lids of the *tankas*

S. no	Anopheles species	Intervention village (Ajasar)			Non-Intervention village (Total)		
		Pre-Intervention survey May/2018	Post- Intervention survey June/2018	Follow-up survey May/2019	Pre-Intervention survey May/2018	Post- Intervention survey June/2018	**Follow-up survey May/2019**
1	*An. culicifacies*	5.1	0.2	0.7	4.1	1.7	1.1
2	*An. stephensi*	10.9	0.6	0.2	11.8	4.6	5.9
3	*An. subpictus*	0.7	0.3	0.6	0.4	2.4	1.1
4	*An. annularis*	0.2	0.0	0.2	0.0	0.0	0.0
Total		16.8	1.1	1.7	16.4	8.1	8.1

### Results of the KAP study in the intervention village

A house-to-house survey was conducted before and after the lids' replacement, with questions about malaria breeding sites, mosquito larvae detection, and other topics. A total of 120 people from different households took part in the survey. The pre-intervention results showed that 67% (80/120) respondents were aware of malaria disease and 37% (44/120) knew that malaria spreads through the bites of female mosquitoes. Only 11% (13/120) respondents were aware that larvae were a part of a mosquito’s life cycle, whereas 20% (24/120) of the respondents knew that mosquitoes breed in *tankas*. In follow-up conducted after one year, there was a significant improvement in the knowledge of respondents (n = 120). The awareness about malaria increased from 67 to 89% (Ҳ^2^ = 17.65, significant at p < 0.001); knowledge about malaria spread through the bite of female mosquitoes improved from 37 to 68% (Ҳ^2^ = 24.13, significant at p < 0.001); knowledge about larval form as a part of mosquito life cycle improved from 11 to 78% (Ҳ^2^ = 110.65, significant at p < 0.001); and the awareness about the breeding of mosquitoes in *tankas* from 20 to 74% (Ҳ^2^ = 70.66, significant at p < 0.001).

## Discussion

As per the National Framework of Malaria Elimination in India 2016–2030, the state of Rajasthan is classified in category 1 because of the overall low API of the state being < 1/1000 [13]. Western Rajasthan, which was an unstable malaria zone before the establishment of the Indira Gandhi Canal in 1970, malaria has become a regular feature in its command areas, indicating a change in the malariological scenario [14].

Since the local population faced scarcity of potable water in this arid zone, they practised stringing rainwater in *tankas* for year-round use. *Anopheles stephensi* had adapted for breeding in *tankas* and *beris* (step wells) throughout the year [7]. Almost all houses in western Rajasthan use *tankas* for their daily water needs and long-term storage. *Tanka* is an important feature that improves the self-reliance of rural people, stabilizes rural employment skills, and is cost-effective [6]. With the increase in water supply through the Indira Gandhi Canal, the resident population has increased in just a few decades, and the number of *tankas* increased, providing enhanced mosquito breeding potential and increased risk of malaria transmission. Availability of water and improper lids allowed mosquito breeding in these *tankas*.

The PVC lids had a low cost (~ INR 400–500) as compared with other lids made up of iron (~ INR 700), aluminium (~ INR 1000) and wood (~ INR 600–1000). Normally, the wooden lids are used as a temporary arrangement to cover the open holes in the *tankas* and their cost may vary according to the quality used.

*Anopheles stephensi,* which is the major malaria vector of western Rajasthan, predominantly breeds in these *tanka* [7]. Community pre-awareness study (KAP) revealed that about 67% of individuals were aware of malaria as a disease spread by mosquitoes and about 44% knew it spread due to infective bites of female *Anopheles*. This shows a fair awareness of the community about the disease and its transmitting agent. However, 13% of the community did not know much about mosquito larvae and only 20% knew that breeding occurs in *tankas* (Fig. 5). Similar results were observed in a study conducted by Yadav et al*.* [15], which also showed that about 20% of respondents know that *tanka* is the main source of mosquito breeding and its proper coverage is essential in the prevention of mosquito breeding [16]. The results showed a significant improvement in the knowledge of the community (22% to 67%) about malaria, its transmission and breeding sites. The significant improvement may be due to the active engagement of villagers in the lid replacement process during the study. The knowledge gained by the community will help understand the importance of proper lids and mosquito proofing of *tankas*.

Damaged lids provide an opportunity for *Anopheles stephensi* to breed in these *tankas*. During hot summer seasons, when outdoor temperatures exceed 45 °C, the *tankas* become resting places for the mosquitoes to avoid desiccation. During the monsoon and post-monsoon seasons, *Anopheles stephensi* breeds profusely and boosts malaria transmission along with another vector species, i.e. *Anopheles culicifacies. Anopheles culicifacies* is a seasonal malaria vector of the Western Rajasthan that appears in the post-monsoon and winter seasons only. A study by Joshi et al*.* showed that *Anopheles stephensi* is a persistent malaria vector in the region and is supported by *Anopheles culicifacies* in the post-monsoon season [17].

Installation of improved polyvinyl lids has completely stopped breeding in these *tankas*. As a result, there was a drastic reduction in the density of *Anopheles stephensi* (94.9% in the post-intervention and 97.9% in the follow-up survey after one year) in the intervention village, Ajasar, as compared to the non-intervention village, Tota. After one year of installation of improved lids their fitness was checked in May 2019. A total of 98% (196/200) replaced lids were found fit and *tankas* were found free from breeding. Furthermore, they had no rusting at hinge joints except for 4 lids, where two lids were found detached from cement binding, probably due to the use of lids before cement settling, and two had mosquito breeding, possibly due to holding lids open for a long period. There was some reduction in *Anopheles stephensi* density (11.8 to 5.9 PMHD) in the non-intervention village (Tota) as well during the post-intervention and follow-up surveys, which may be attributed to the impact of IEC on villagers about the importance of lids and mosquito breeding.

According to the World Malaria Report 2019, almost 85% of the global malaria burden is distributed in nineteen countries in Sub-Saharan Africa and India [18]. India accounted for 47% of the *Plasmodium vivax* burden. Seven states of India viz. Jharkhand, West Bengal, Uttar Pradesh, Chhattisgarh, Odisha, Gujarat and Madhya Pradesh contributed a combined 90% of malaria cases. In Rajasthan, the incidence of *Plasmodium falciparum* was very low i.e. 8% of total malaria cases in Rajasthan in 2016, which increased to 13% in 2020. Although malaria cases have declined from 12,741 in 2016 to 674 in 2020 (up to September), the *Plasmodium falciparum* percentage increased [19]. The availability of vectors like *Anopheles stephensi* during the immigration of such people coming to the village either post-monsoon for crop purposes or for developmental activities might introduce parasites into the village system for transmission [17].

## Conclusion

The current study has shown that small local interventions can efficiently solve an emerging problem of malaria. A significant reduction in the density of *Anopheles stephensi* was observed using mosquito-proof lids, addressing the problem of persistent mosquito breeding in underground tanks. The modified polyvinyl plastic lids are of low cost and have a life expectancy of approximately 2 to 5 years if used properly. Lids not only prevent the entry of mosquitoes in *tankas,* but also of other opportunistic insects and reptiles such as lizards, snakes, and rodents.

Rajasthan is a low malaria transmission area, falling under category 1 of the WHO’s malaria elimination target for 2030. These novel measures could help to curb perennial malaria transmission by reducing the responsible vector, which may be responsible for year-round malaria transmission. Such simple long-term interventions are critical because they reduce routine intervention costs, reduce insecticide waste, and protect the environment.

## Abbreviations

ANM: Auxiliary Nurse Midwife
API: Annual Parasite Index
BDO: Block Development Officer
CHC: Community Health Center
GPS: Global Positioning System
IEC: Information, Education and Communication
KAP: Knowledge, Attitude and Practices
NVBDCP: National Vector Borne Disease Control Program
PMHD: Per Man Hour Density
PVC: Polyvinyl chloride
WHO: World Health Organization
